# The Phytochemical Bergenin Enhances T Helper 1 Responses and Anti-Mycobacterial Immunity by Activating the MAP Kinase Pathway in Macrophages

**DOI:** 10.3389/fcimb.2017.00149

**Published:** 2017-05-01

**Authors:** Ved P. Dwivedi, Debapriya Bhattacharya, Vinod Yadav, Dhiraj K. Singh, Santosh Kumar, Mona Singh, Durbadal Ojha, Anand Ranganathan, Luc Van Kaer, Debprasad Chattopadhyay, Gobardhan Das

**Affiliations:** ^1^Immunology Group, International Centre for Genetic Engineering and BiotechnologyNew Delhi, India; ^2^Special Centre for Molecular Medicine, Jawaharlal Nehru UniversityNew Delhi, India; ^3^Department of Microbiology, Central University of HaryanaMahendergarh, India; ^4^Regional Medical Research CentreBelagavi, India; ^5^Department of Pathology, Microbiology and Immunology, Vanderbilt University School of MedicineNashville, TN, USA

**Keywords:** *Mycobacterium tuberculosis*, T cells, immunomodulation, bergenin, vaccine

## Abstract

Tuberculosis (TB) remains one of the greatest health concerns worldwide, which has hindered socioeconomic development in certain parts of the world for many centuries. Although current TB therapy, “Directly Observed Treatment Short-course,” is effective, it is associated with unwanted side effects and the risk for the generation of drug-resistant organisms. The majority of infected individuals successfully confine the mycobacterial organisms and remain asymptotic unless immune responses are perturbed. Thus, host immunity can protect against TB and immunomodulation is therefore an attractive therapeutic option. Previous studies have shown that TNF-α and Nitric Oxide (NO) in conjunction with IFN-γ-producing T helper 1 (Th1) cells play critical roles in host protection against TB. Here, we show that bergenin, a phytochemical isolated from tender leaves of *Shorea robusta*, activates the MAP kinase and ERK pathways and induces TNF-α, NO and IL-12 production in infected macrophages. We further show that bergenin induces Th1 immune responses and potently inhibits bacillary growth in a murine model of *Mycobacterium tuberculosis* infection. These findings identify bergenin as a potential adjunct to TB therapy.

## Introduction

Tuberculosis (TB) is a global health problem, claiming two million lives every year (WHO, 2015b). TB is the oldest known human infectious disease, yet an effective and reliable vaccine or therapy are not available. The only vaccine employed, Bacillus Calmette Guerin (BCG), was developed in 1921, but fails to protect against adult pulmonary TB (Fine, 1989, 1995; Udani, 1994; Colditz et al., 1995; Brewer, 2000; Simona and Traian, 2013; WHO, 2015a). Current therapy for TB, adopted as the internationally recognized “Directly Observed Treatment Short-course (DOTS),” consists of mutiple toxic antibiotics and involves a lengthy regimen associated with significant risk for the generation of drug-resistant organisms (Davies, 1996; Byrd and Davis, 2007). Most countries, irrespective of their socioeconomic status, are under threat from attack by multiple and extremely drug-resistant (MDR and XDR) *strains of Mycobacterium tuberculosis* (*M.tb;* Byrd and Davis, 2007; Davies, 1996). Furthermore, these antibiotics exhibit hepatic and immune toxicity, with the potential to cause hepatitis and to eliminate antigen-specific T cells (Fountain et al., 2005; Cox et al., 2008). The latter complication may result in hypersusceptibility to disease reactivation and reinfection following treatment (Iseman, 1993; Cox et al., 2008). Therefore, an alternate therapeutic strategy that avoids these limitations is urgently needed to combat this deadly organism.

Following infection, the host immune system typically confines the organism and only a fraction (~10%) of individuals infected with *M.tb* progress toward TB disease (Flynn and Chan, 2001). Both mice and humans with genetic defects in IFN-γ signaling are highly susceptible to mycobacterial diseases (Flynn and Chan, 2001). It is now well-established that IFN-γ-producing CD4^+^ T cells play important roles in host protection against TB (Flynn and Chan, 2001; Sweeney et al., 2011). Consequently, mice deficient in interleukin (IL)-12, the IL-12 receptor, signal transducers of activation and transcription factor (STAT)-4, and T-bet, each of which exhibits defective Th1 immune responses, are highly susceptible to *M.tb* infection (Lienhardt et al., 2002; Cooper et al., 2007). It has been well-documented that *M.tb* inhibits Th1 cell differentiation by dampening IL-12 production in infected macrophages (Hickman et al., 2002; Trinchieri, 2003; Cooper et al., 2007). On the other hand, regulatory T cells (Tregs) and IL-4-producing Th2 cells have been shown to assist in disease progression by inhibiting Th1 cell responses (Kursar et al., 2007; Scott-Browne et al., 2007; Chen and Konkel, 2010; Shafiani et al., 2010; Yoshimura et al., 2010). The role of IL-17-producing Th17 cells during primary infection is less well-understood (Chatterjee et al., 2011), but its role in protective immunity against re-infection and its importance for vaccine efficacy are well-documented (Chatterjee et al., 2011). All Th cell subsets, including Th1, Th2, Th17, and Treg cells are in a dynamic balance, and hence elevation of Th1 responses assists bacterial clearance not only by enhancing cell-mediated immune responses but also by inhibiting unwanted humoral immune responses (Flynn and Chan, 2001). Consequently, immunotherapy may be preferable over antibiotic treatment for avoiding the generation of MDR and XDR strains of *M.tb*.

In order to identify novel immunomodulatory agents with therapeutic potential against TB we have focused on ethnomedical agents, which are typically well-tolerated by patients. In our efforts to characterize such compounds, we identified a large number of ethnomedical compounds that showed potent immunomodulatory activity (Chattopadhyay et al., 2012; Mukherjee et al., 2013; Jnawali et al., 2016; Lee and Suh, 2016; Gomez-Cansino et al., 2017). One of these compounds is bergenin, a tri-hydroxybenzoic acid or C-glycoside of 4-O-Methyl Gallic acid (Stanford et al., 1990; Narita et al., 1998; Patel et al., 2012) known as Paashaanbhed in Ayurveda. This is a well-tolerated compound in humans whose potential utility for treatment of infectious diseases has not been previously investigated. We found that bergenin has potent immunomodulatory activity and selectively induces IFN-γ- and IL-17-producing phenotypes in both CD4+ and CD8+ T cells. Consequently, bergenin potently enhanced clearance of *M.tb* from infected mouse hosts. Mechanistically, we found that these effects of bergenin on Th cell responses were due to induction of MAPK, ERK1/2, and SAPK/JNK pathways in infected macrophages. Collectively, our findings suggest that addition of bergenin to conventional antibiotic therapy may promote clearance of *M.tb* organisms in patients.

## Results and discussion

### Immunomodulatory activity of bergenin *In vitro*

**Figure 1:**
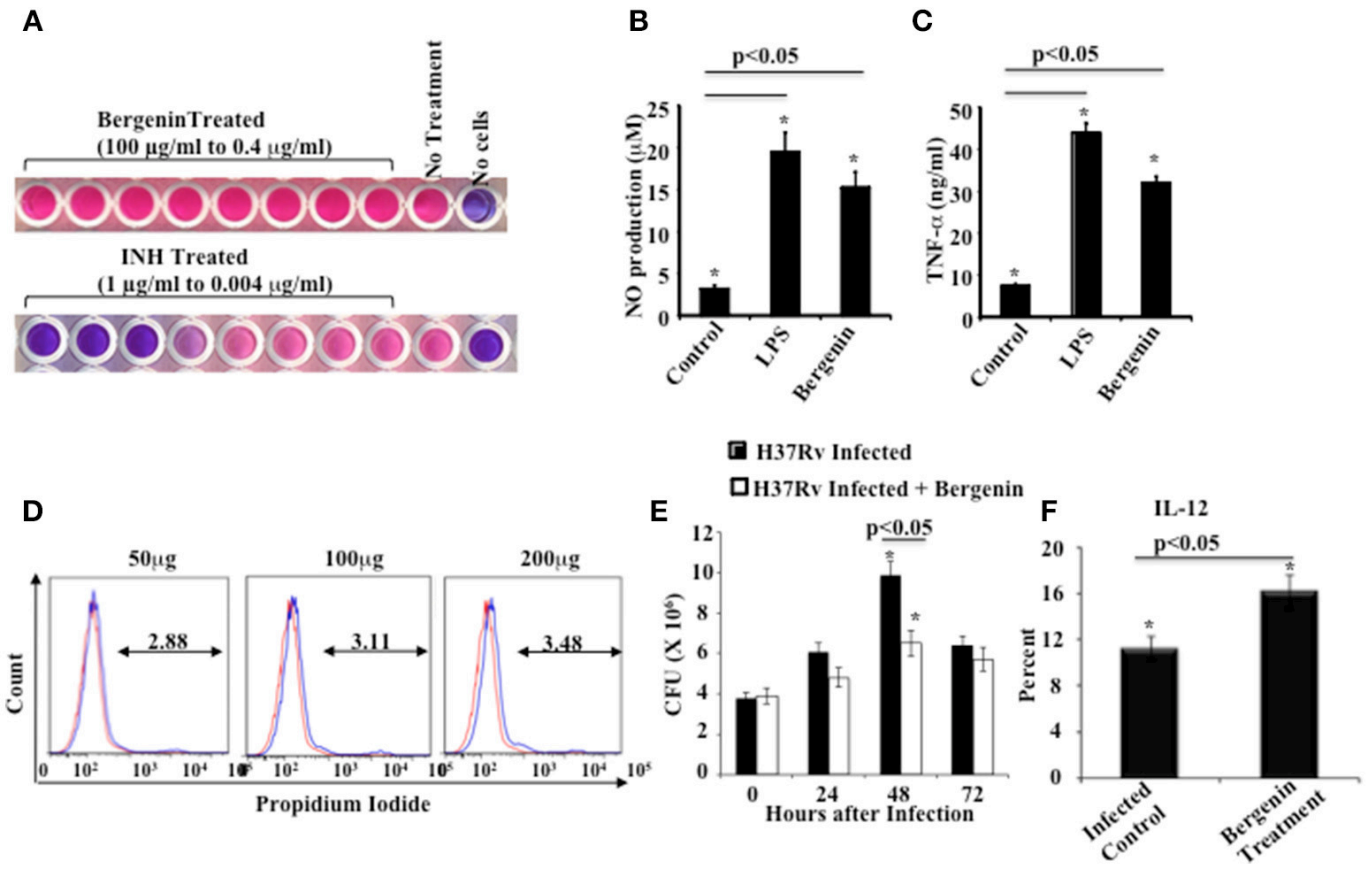
*****In vitro*** effects of bergenin on macrophages. (A)** Anti-mycobacterial activity of bergenin and isoniazid (INH) as assessed by Alamar Blue assay. **(B,C)** Measurement of NO **(B)** and TNF-α **(C)** production by macrophages after bergenin (50μg/ml) or LPS (1μg/ml) treatment. **(D)** Propidium iodide assay for cell viability following treatment of macrophages with graded concentrations of bergenin. The red line represents untreated macrophages. **(E)** Bacterial survival in macrophages infected with H37Rv and treated with or without bergenin. **(F)** Percentage of IL-12 production with or without Bergenin treatment. The results shown are representative of four independent experiments. Error bars indicate means ± SD. ^*^p < 0.05.

We aimed to bias Th cell responses against *M.tb* using a novel immunomodulator. From the vaccine studies it is clear that Th1 responses are essential but not sufficient for host protection against TB (Bhattacharya et al., 2014a,b). Therefore, we screened a variety of plant-based compounds for anti-mycobacterial as well as immunomodulatory activity. Ethnomedical drugs are typically well-tolerated and possess potent efficacy against a variety of diseases (Staub et al., 2015). Such natural products with medicinal properties are often extracts from natural herbs and spices that include one or more active compounds. We screened ethnomedical compounds for reagents that enhance the therapeutic efficacy of ATT. We selected bergenin, a C-glycoside of 4-O-methyl Gallic acid (Stanford et al., 1990; Narita et al., 1998; Patel et al., 2012), for further analysis and found that it exhibits potent immunomodulatory activity. Its potential efficacy against infectious diseases has not been previously tested. Our initial goal was to determine whether bergenin has any antimicrobial activity. For this purpose we performed an ALAMAR Blue assay. We treated *M.tb* strain H37Rv with bergenin and used isoniazid as a positive control. Results indicated that bergenin lacks direct anti-mycobacterial activity (Figure 1A). Next we evaluated effects of bergenin on host immune cells. We incubated murine macrophages with bergenin and measured the production of NO and TNF-α. Interestingly, bergenin induced the secretion of NO (Figure 1B) as well as TNF-α (Figure 1C). Cell viability assays showed that bergenin does not show any cytotoxicity against macrophages (Figure 1D). To examine the effect of bergenin on bacterial loads in infected cells we performed *in vitro* Colony Forming Unit (CFU) assays. We infected macrophages with H37Rv, treated them with bergenin and measured CFUs at various time points thereafter. We found that bergenin treatment significantly reduces the bacterial burden (Figure 1E). These data were further strengthened by profiling of IL-12 levels in the macrophages after H37Rv infection with or without bergenin treatment (Figure 1F). Collectively these data suggested that bergenin lacks direct anti-mycobacterial activity but indirectly kills *M.tb* in infected macrophages.

### Bergenin enhances *M.tb* specific immune responses *In vivo*

**Figure 2:**
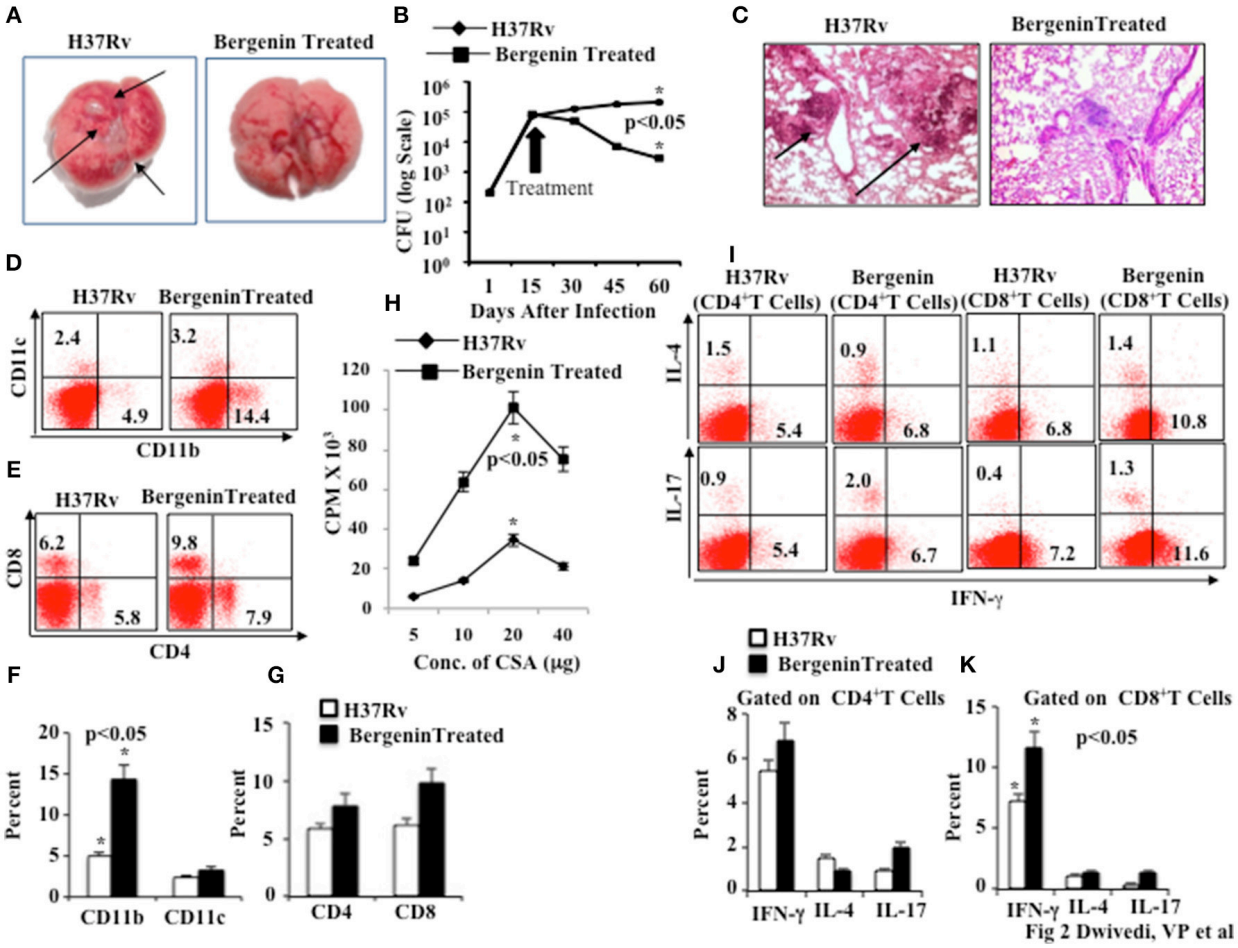
**Immunomodulatory effect of bergenin on mice infected with ***Mycobacterium tuberculosis***. (A)** Macroanatomic pictures to show effects of bergenin on the lungs of H37Rv-infected mice. Arrows indicate the lesions in the lung of *M.tb* infected mice. **(B)** CFU from the lung homogenates of mice that were infected with H37Rv and treated with bergenin. **(C)** Histology pictures to show effects of bergenin on infection. Arrows indicate the granulomatic lesions in the lung section of *M.tb* infected mice. **(D,F)** FACS data to show the percentage of dendritic cells (CD11c+) and macrophages (CD11b+). **(E,G)** FACS data to show the percentage of CD4+ and CD8+ T cells. **(H)** Proliferation of splenocytes isolated from H37Rv-infected and bergenin-treated mice in response to CSA was measured by [3H]-thymidine incorporation assay. **(I–K)** Intracellular staining for IFN-γ, IL-4, and IL-17 of CD4+ and CD8+ T cells isolated from the spleen of H37Rv-and bergenin-treated mice. The results shown are representative of three independent experiments with six mice within each group. Error bars indicate means ± SD. ^*^p < 0.05.

Next, we assessed the *in vivo* efficacy of bergenin against TB in a murine model of *M.tb* infection. We infected C57BL/6 mice with a low dose (~110 CFU) of *M.tb* H37Rv through the aerosol route. At various time points after infection we harvested lungs and spleen for determination of bacterial loads. We found that bergenin treatment significantly reduces the number of granulomatic lesions as shown in macroanatomic pictures of the lungs (Figure 2A), and bacterial burdens were about 2 logs lower than in untreated animals (Figure 2B). These findings suggested that adaptive immune responses play an important role in the capacity of bergenin to enhance bacterial clearance. These data were further strengthened by histological analyzes of the lungs, which revealed reduced numbers of granulomas after bergenin treatment (Figure 2C).

Next, we immune phenotyped different immune cells in the spleens of bergenin-treated animals. We found that bergenin treatment significantly increases the prevalance of macrophages (CD11b^+^ cells) and dendritic cells (CD11c^+^ cells; Figures 2D,F), which are the main APCs that play an important role in the induction of adaptive immune response. We also found an increase in the prevalence of CD4^+^ T and CD8^+^ T cells in the spleen (Figures 2E,G) and lungs (Figures S1A,B) after bergenin treatment. Next, we investigated *M.tb* antigen-specific T cell proliferation in the splenocytes from infected animals and found that bergenin treatment significantly enhances proliferation of antigen-specific T cells (Figure 2H). To investigate effects on cytokine production by T cells we performed intracellular staining of characteristic Th1, Th2, and Th17 cytokines. We found that bergenin treatment enhances the frequency of IFN-γ- and IL-17-producing T cells, whereas no significant differences were observed for IL-4-producing T cells (Figures 2I–K). Taken together, our data suggest that bergenin promotes the generation of protective immune responses against TB.

### Bergenin induces the MAPK, ERK, and SAPK/JNK pathways in macrophages

**Figure 3:**
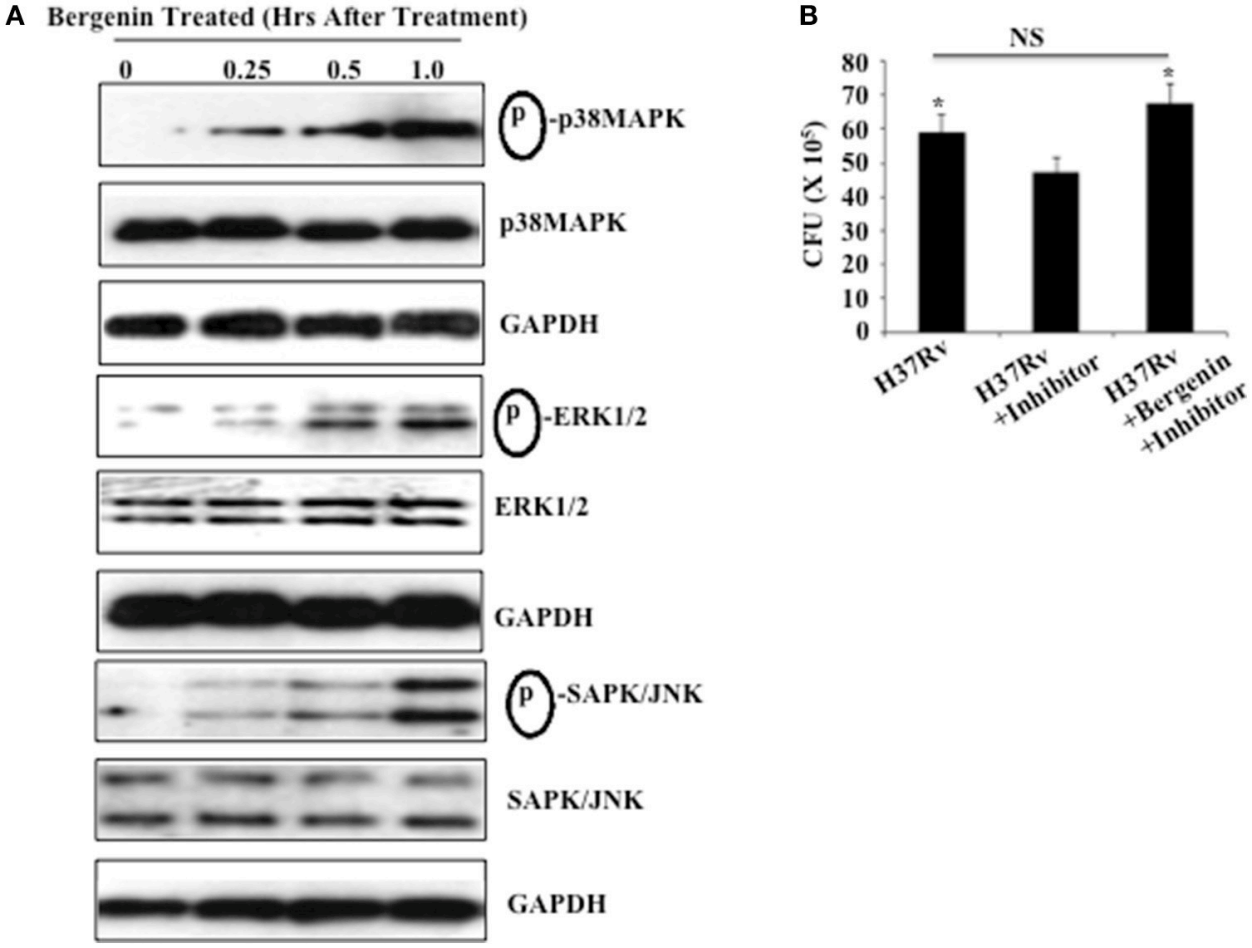
**Bergenin treatment activates the MAPK pathway. (A)** Peritoneal macrophages were untreated or treated with 50-μg/ml bergenin for different time periods. Phosphorylation of p38 MAPK, ERK1/2, and SAPK/JNK was assessed in whole cell lysate of bergenin-treated or untreated macrophages by Western blot. **(B)** Bacterial burden in the macrophages after treatment with MEK-1/2 inhibitor (U0126 from Cell Signaling Technologies). We isolated peritoneal macrophages, treated these cells with a MEK-1/2 inhibitor (20 μM) overnight, followed by infection with H37Rv and treatment with or without bergenin. Forty-eight hours later we harvested the cells for CFU assay, which revealed that treatment with bergenin was unable to reduce the bacterial burden in the presence of the MEK-1/2 inhibitor. The results shown here are representative of three independent experiments. ^*^Indicates significant for *p*-value.

Because bergenin induces production of NO and TNF-α by macrophages and reduces the bacterial load in these cells, we investigated its effects on MAPK signaling in macrophages, which is critically important for the induction of pro-inflammatory cytokines (Whitmarsh et al., 1997; Zarubin and Han, 2005). Peritoneal macrophages derived from C57BL/6 mice were treated with bergenin (50 μg/ml) for varying time points and activation of MAPK pathways (p38 MAPK, ERK1/2, and SAPK/JNK) was investigated via Western blotting (Figure 3). Relative to untreated macrophages a time-dependent increase in the phosphorylation of p38 MAPK, ERK1/2, and SAPK/JNK was observed in bergenin-treated macrophages. Notably, non-phosphorylated p38 MAPK, ERK1/2, SAPK/JNK, and GAPDH levels were unaltered following bergenin treatment (Figure 3A). In order to determine the contribution of MEK-1/2 we isolated peritoneal macrophages, treated these cells with an MEK-1/2 inhibitor (U0126 from Cell Signaling Technologies) overnight, followed by infection with H37Rv and treatment with or without bergenin. Forty-eight hours later we harvested the cells for CFU assay, which revealed that treatment with bergenin was unable to reduce the bacterial burden in the presence of the ERK inhibitor (Figure 3B). Therefore, these findings suggest that bergenin can target the MAP kinase pathway to alter host immune responses.

## Conclusions

*M.tb* survives within susceptible hosts by altering host protective Th cell responses. A key mechanism in protective immunity against TB is IFN-γ-induced production of NO radicals by macrophages (Dwivedi et al., 2012). Thus, animals that are defective in the production of IL-12, inducible NO synthase, IFN-γ, or proteins involved in their signaling pathways exhibit increased susceptibility to *M.tb* infection (Dwivedi et al., 2012). In addition, it has been shown that *M.tb* not only prevents Th1 responses, but also facilitates Th2 responses (Rook, 2007; Dwivedi et al., 2012; Bhattacharya et al., 2014a,b) that oppose host protective Th1 responses. Phagocytes, where *M.tb* organisms replicate, are the predominant source of IL-12 that drives Th1 cell differentiation (Flynn et al., 1993; Rahman et al., 2015). On the other hand, Th2 cells and Tregs facilitate disease progression by inhibiting host protective Th1 responses. Interestingly, Th17 cells play a crucial role in host resistance during secondary infections (Chatterjee et al., 2011). Recently, we have shown that simultaneous inhibition of Th2 cells and Tregs significantly reduces tubercular burden in infected animals but is unable to completely clear *M.tb* organisms from the host (Bhattacharya et al., 2014a,b). Therefore, we screened several plant-based compounds for protective immunity against *M.tb* infection. We selected one compound, bergenin, for further analysis. Bergenin exhibits anti-hepatotoxic, anti-ulcerogenic, anti-HIV, anti-arrhythmic, neuroprotective, anti-inflammatory, and immunomodulatory properties (Patel et al., 2012). Mechanistically, bergenin mediates some of its beneficial properties by protecting cells against oxidative stress (Patel et al., 2012). For example, the free radical scavenging activity of bergenin protects against radiation-induced liposomal lipid peroxidation, protein carbonylation and DNA damage (Patel et al., 2012). In our studies bergenin did not exhibit direct anti-mycobacterial activity, but showed potent immunomodulatory activity, induced production of NO and TNF-α in macrophages, and reduced the bacterial burden of infected cells. *In vitro* bergenin induced cytokines in macrophages that created an environment conducive to killing microbes, but this was insufficient for effective clearance of the bacilli. However, in a murine model of *M.tb* infection bergenin induced macrophage and dendritic cell expansion and promoted the generation of *M.tb* specific adaptive immune responses. Thus, *in vivo* bergenin appears to activate not only macrophages but also other cell types such as dendritic cells and T cells that contribute to the effective killing of bacteria, providing a potential explanation for the differences observed in our *in vitro* and *in vivo* studies.

We found that bergenin promotes the expansion of protective Th1 and Th17 responses, with limited effects on Th2 responses. Additionally, we showed that bergenin enhances activation of the MAP kinase pathway in infected macrophages, which is critically important for the induction of pro-inflammatory cytokines (Whitmarsh et al., 1997; Zarubin and Han, 2005). The activation of MAP kinases in macrophages provides protective host immunity during *M.tb* infection and produces various effector molecules that show anti-mycobacterial activity. For example, ERK induces secretion of iNOS and NO in macrophages (Chan et al., 2001). However, *M.tb* contains immune evasion mechanisms to limit the activity of MAP kinases (Schorey and Cooper, 2003). Activation of MAP kinases by bergenin may counteract these mycobacterial immune evasion mechanisms.

Taken together, our data suggest that bergenin, a potent immunomodulatory compound, induces strong protective Th1 and Th17 cell responses and drastically reduces the mycobacterial burden in infected animals. One of our future goals is to employ bergenin as an adjunct therapy with ATT (anti-tuberculosis therapy) that can possibly reduce the length of treatment, toxicity associated with ATT, re-infection and disease reactivation, and emergence of drug-resistant bacterial variants. Moreover, bergenin holds promise as an adjuvant to TB vaccines.

## Materials and methods

### Ethics statement

Animal experiments were performed according to the guidelines approved by the Institutional Animal Ethics Committee of the International Centre for Genetic Engineering and Biotechnology (ICGEB; New Delhi, India) and the Department of Biotechnology guidelines (government of India). All mice used for experiments were ethically sacrificed by asphyxiation in carbon dioxide according to institutional and Department of Biotechnology, Government of India, regulations.

### Mice

C57BL/6 (6–8 wks of age) mice were provided by our institute (ICGEB, New Delhi, India). All animals were maintained in the animal facility of the ICGEB.

### Bacteria

*M.tb* strain H37Rv was a kind gift from the Colorado State University repository. Organisms were grown in 7H9 (Middlebrooks, Difco™, USA) medium supplemented with 10% ADC (albumin, dextrose, and catalase; Difco™, USA) and with 0.05% Tween 80 and 0.2% glycerol, and cultures were grown to mid-log phase. Aliquots of the cultures in 20% glycerol were preserved at −80°C and these cryo-preserved stocks were used for infections.

### Isolation of compound(s)

Young or tender leafs of *Shorea robusta* L. were collected throughout the year from the nearby forest of the tribal area of Hazaribagh (Jharkhand, India). The specimens were identified and authenticated by a taxonomist from the Botanical Survey of India (Shibpur, Howrah, India). For isolation of active compound(s), a methanol extract was partitioned between water-saturated *n*-butanol and water, and the organic part was concentrated under reduced pressure to a dark brown residue (98 g). The residue was chromatographed on silica gel (60–120 mesh) column, and graded elution was carried out with chloroform (100%) and a mixture of chloroform-methanol at a ratio of 95:5, 90:10, and 80:20, and collected as fractions. Out of 176 sub-fractions (200 ml each) 6 major fractions were collected, based on similar spots on TLC. These fractions were successively re-chromatographed with graded chloroform (100%) followed by a mixture of chloroform-methanol at a ratio of 99:1, 97:3, 95:5, 93:7, 90:10, 85:15, and 80:20 and were segregated by TLC into three fractions, of which fraction 2 and 3 showed significant antimicrobial activity. The re-chromatograph of fraction 2, eluted with CHCl_3_–MeOH (95:5) and CHCl_3_–MeOH (80:20) over silica gel (100–200 mesh) resulted in a colorless solid (Compound **1**, yield ≈ 0.075%); whereas fraction 3 yielded a pale yellow crystallized micro-needle (Compound **2**, yield ≈0.059%). Chemical characterization was performed by IR (JASCO7300 FTIR spectrometer), Mass (Q-TOF Micromass Spectrometer), ^1^HNMR (at 600 and 300 MHz), and ^13^CNMR (at 150 and 74.99 MHz) by Bruker AVANCE 600 spectrometer with TMS as internal standard in C_5_D_5_N and/or MeOD. The melting points (m.p.) were measured on a Yanagimoto Micro melting Point Apparatus, whereas JASCO DIP-370 digital polarimeter was used for the detection of optical rotations.

### Characterization of the isolated compound

The IR spectra of a crystallized colorless micro needle, obtained from methanol extract with melting point 236°C, showed absorption bands at 3,391, 1,704, 1,462 Cm^−1^ due to hydroxy and keto groups, and aromatic double bonds. The quasi-molecular ion peak [M+Na]^+^ at m/z 351 revealed its molecular weight to be 328. This information coupled with the data from ^13^C NMR and DEPT spectra suggested the molecular formula as: C_14_H_16_O_9_. Further, a signal at δ 7.70 (^1^H) in ^1^H NMR spectra was due to an aromatic proton, while the three-proton sharp singlet at δ 3.99 indicated an aromatic methoxy group; a singlet at δ 164.7 was due to a COOH group, while a signal at δ 62.8 signifies CH_2_OH group; and a methyl peak at δ 60.5 confirmed the methoxy group, along with six methine signals at δ 111.3, 83.7, 81.6, 75.8, 74.1, and 72.4. Thus, collectively these data suggested that the Compound **1** was bergenin (Mukherjee et al., 2013).

### Alamar blue assay

Bacilli were grown until mid log phase (OD_600_ 0.6–0.8) and added to a 96-well plate containing two-fold serial dilutions of bergenin (1 μg/ml) at final OD_600_ of 0.01. Isoniazid (1 μg/ml) was used as a positive control. After incubation at 37°C for 5 days 1X alamar blue (Thermo Fisher Scientific) was added in each well and a color change from blue to pink was observed after 16 h of incubation. Wells containing only media and no bacilli served as negative control and wells having only bacilli without any drug were treated as positive control.

### *M.tb* infection of mice and estimation of colony forming units (CFU)

Mice were infected with *M.tb* H37Rv via the aerosol route using a Madison aerosol chamber (University of Wisconsin, Madison, WI) with its nebulizer pre-calibrated to deposit a total of ~110 CFU to the lungs of each mouse as previously described (Chatterjee et al., 2011). Briefly, mycobacterial stocks recovered from a −80°C freezer were quickly thawed and subjected to light ultrasonication to obtain a single cell suspension. Fifteen milliliters of the bacterial cell suspension (10 × 10^6^ cells per ml) was placed in the nebulizer of the Madison aerosol chamber pre-calibrated to deliver via aerosol route the desired number of CFUs to the lungs of animals placed inside the chamber. One day after the aerosol exposure procedure, three randomly selected mice were sacrificed at various time points and organs were harvested, homogenized in 0.2 μm filtered PBS containing 0.05% Tween 80 and plated onto 7H11 Middlebrooks (Difco USA) plates containing 10% oleic acid, albumin, dextrose, and catalase (OADC) (Difco, USA). Undiluted, 10-fold diluted and 100-fold diluted lung and spleen cell homogenates were plated in duplicate on the above 7H11 plates and incubated at 37°C for 21–28 days. Colonies were counted and CFU were estimated. Mice from various groups were euthanized at the indicated time points in various experiments and their organs were harvested for obtaining CFU counts and/or immune cell subpopulations for immunological studies as described under other sub-sections.

### Drug administration

Four milligrams or kilograms of bergenin in 100 μl of PBS was administered intraperitoneally every day during the entire treatment phase.

### Isolation of mouse peritoneal macrophages

Six- to eight-week-old female C57BL/6 mice were given an *i.p*. injection of 2 ml thioglycollate medium (4%). Five days after injection macrophages were obtained by peritoneal lavage. Macrophages were washed once with cold PBS and re-suspended in cold RPMI-1640 and counted by Trypan blue exclusion method. Cells (0.5 × 10^6^ per well) were seeded on 12-well plates and maintained at 37°C in RPMI-1640 medium supplemented with or without penicillin–streptomycin (1,000 units/ml) and 10% heat-inactivated fetal calf serum. After overnight incubation, non-adherent cells were washed and the purity of the adherent macrophages was determined by flow cytometric analysis using anti-CD11b antibody, which suggested that >95% of the cells in the peritoneal lavage were macrophages.

### Determination of cytotoxicity

Monolayers of macrophages in 96-well tissue culture plates (1.0 × 10^5^ cells/ml) were grown and incubated at 37°C in 5% CO_2_ for 6 h, different concentrations of test compound were added to culture wells in triplicate at a final volume of 100 μl, and incubated for 48 h. Thereafter, the medium was replaced with fresh RPMI containing 1 mg/ml of Propidium iodide. Cells were incubated at 37°C for 15 min and then washed with PBS and acquired and analyzed with a FACS Canto II flow cytometer (BD Biosciences, USA).

### FACS analysis

For intracellular cytokine staining, cells were treated with 50 ng/ml PMA and 500 ng/ml ionomycin in the presence of 10 μg/ml brefeldin A (Sigma-Aldrich or e-Biosciences, USA) added during the last 6 h of culture. Cells were washed twice with PBS, resuspended in a permeabilization buffer (Cytofix/Cytoperm kit; BD), and stained with the following fluorescently conjugated monoclonal antibodies: anti-CD4 (clone GK1.5)-allophycocyanin (APC), anti-IFN-γ (clone XMG1.2)-fluorescein isothiocyanate (FITC), anti-IFN-γ (clone XMG1.2)-APC, anti-IL-4 (clone GK1.5)-phycoerythrin (PE) (e-Biosciences, USA). Fluorescence intensity was measured by flow cytometry (FACS Canto II; BD) and data were analyzed with Flow Jo (Tree star, USA).

### [^3^H]-thymidine incorporation assay of splenocytes

Spleens from C57BL/six mice were macerated by frosted slides in complete RPMI-1640 (Gibco, Invitrogen, UK) and made into a single cell suspension. Red blood cells (RBCs) were lysed with RBC cell lysis buffer and washed with complete RPMI-1640. Splenocytes were counted and plated at 0.1 × 10^6^ cells/well in 96-well plates and stimulated with different concentrations of *M.tb* complete soluble antigen (CSA). Cells were cultured for 48 h and then pulsed with tritiated thymidine (^3^H-TdR, 1.0 μCi per well; Amersham Biosciences UK) before measuring incorporation of ^3^H-TdR by means of a cell harvester and liquid scintillation counter 16 h later (Perkin Elmer, UK).

### Western blotting

Peritoneal macrophages derived from C57BL/6 mice were treated with bergenin (50 μg/ml) for 15 min, 30 min, or 1 h. Whole cell lysate was prepared by using lysis buffer (50 mM Tris-HCl, pH 7.4, 5 mM EDTA, 120 mM NaCl, 0.5% Nonidet P-40, 0.5 mM NaF, 1 mM dithiothreitol, 0.5 mM phenylmethylsulfonyl fluoride) along with a phosphatase inhibitor mixture (78,420; Thermo Scientific) and protease inhibitor mixture (78,410; Thermo Scientific) for 1 h. Samples were electrophoresed on a 10% SDS-polyacrylamide gel and electroblotted onto polyvinylidenedifluoride (PVDF) membranes. Blots were blocked for 1 h in 5% BSA in PBST (PBS with 0.1% Tween 20), and p-ERK, ERK, p-P38, P38, p-SAPK/JNK, and SAPK/JNK proteins were detected with anti-ERK, -pERK, -P38, -pP38, and -JNK polyclonal antibodies, respectively, at a dilution as recommended by the manufacturer (Cell Signaling Technologies). Goat anti-rabbit immunoglobulin G-conjugated horseradish peroxidase (diluted 1:2500) was used as a secondary antibody (Cell Signaling Technologies). Immunoblotting for GAPDH was carried out to confirm equal loading.

### Statistical analysis

All data were derived from at least three independent experiments. Statistical analyzes were conducted using SPSS10 software and values were presented as mean ± SD. Significant differences between the groups were determined by ANOVA followed by Tukey's multiple comparison test (SPSS software). A value of *p* < 0.05 was accepted as an indication of statistical significance.

## Author contributions

VD, DB, VY, DS, SK, MS, and DO did the experiments. Isolation, characterization of bergenin was done by DO and DC. VD, AR, DC, LV, and GD wrote the manuscript.

### Conflict of interest statement

The authors declare that the research was conducted in the absence of any commercial or financial relationships that could be construed as a potential conflict of interest.

## Supplementary material

Figure S1 **Effect of bergenin treatment on lung immune cells. (A,B)** Profiling of CD4+ and CD8+ T cells in the lungs of mice infected with H37Rv and treated with or without bergenin.
