# Supplementary figures and images for: The Phytochemical Bergenin Enhances T Helper 1 Responses and Anti-Mycobacterial Immunity by Activating the MAP Kinase Pathway in Macrophages
Source: Front Cell Infect Microbiol. 2017 May 1;7:149. doi: 10.3389/fcimb.2017.00149 (PMC5410567; doi:10.3389/fcimb.2017.00149)

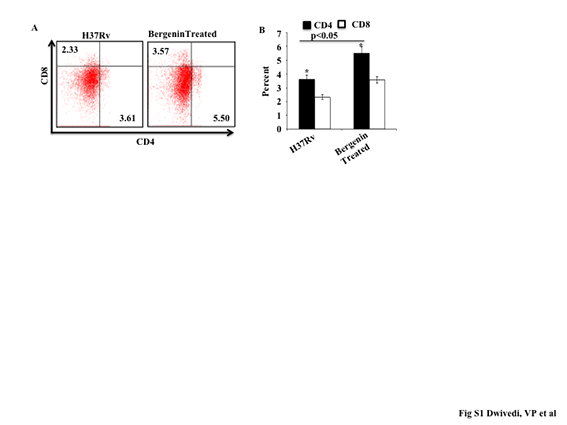

Supplement: Figure S1 — Effect of bergenin treatment on lung immune cells. (A,B) Profiling of CD4+ and CD8+ T cells in the lungs of mice infected with H37Rv and treated with or without bergenin. [file Image1.TIFF]
